# Adapting Simon’s Two-Stage Design for Efficient Screening of Filovirus Vaccines in Non-Human Primates

**DOI:** 10.3390/vaccines10081216

**Published:** 2022-07-29

**Authors:** Nancy A. Niemuth, Carol L. Sabourin, Lucy A. Ward

**Affiliations:** 1 Battelle, Columbus, OH 43201, USA; carol.sabourin@hhs.gov; 2 National Institute of Allergy and Infectious Diseases, National Institutes of Health, Bethesda, MD 20892, USA; lucy.a.ward.civ@army.mil

**Keywords:** vaccine screening, Marburg virus (MARV), Sudan virus (SUDV), Ebola virus (EBOV), Simon’s two-stage

## Abstract

The cynomolgus monkey (*Macaca fascicularis*) non-human primate (NHP) is widely used for filovirus vaccine testing. To use limited BSL-4 resources efficiently and minimize NHP usage, Simon’s two-stage design was adapted to screen candidate Ebola virus (EBOV) vaccines in up to six NHPs with two (optimal), three, or four NHPs in Stage 1. Using the optimal design, two NHPs were tested in Stage 1. If neither survived, the candidate was rejected. Otherwise, it was eligible for Stage 2 testing in four NHPs. Candidates advanced if four or more NHPs were protected over both stages. An 80% efficacious candidate vaccine had 88.5% probability of advancing, and a 40% efficacious candidate vaccine had 83% probability of rejection. Simon’s two-stage design was used to screen 27 EBOV vaccine candidates in 43 candidate regimens that varied in dose, adjuvant, formulation, or schedule. Of the 30 candidate regimens tested using two NHPs in Stage 1, 15 were rejected, nine were withdrawn, and six were tested in Stage 2. All six tested in Stage 2 qualified to advance in the product development pipeline. Multiple regimens for the EBOV vaccines approved by the European Medicines Agency (EMA) and the US Food and Drug Administration (FDA) in 2019 were tested in this program. This approach may also prove useful for screening Sudan virus (SUDV) and Marburg virus (MARV) vaccine candidates.

## 1. Introduction

Filoviruses (Family Filoviridae genera Ebolavirus and Marburgvirus) are negative-stranded RNA viruses known to infect humans and non-human primates (NHPs), causing severe health consequences including death. Human infection occurs as the result of contact with infected individuals or animals with subsequent person-to-person spread. Filovirus infections have resulted in case fatality rates of up to 90% in humans depending on virus strain and clinical care [1]. The sporadic nature of disease outbreaks presents a challenge to evaluating the efficacy of vaccines and other medical countermeasures (MCM) in human clinical trials, which is the standard regulatory path for licensure and approval by the US Food and Drug Administration (FDA) and European Medicines Agency (EMA). Except in outbreak settings, it is likely that vaccines and other MCM against filoviruses will require development and testing in accordance with the FDA Animal Rule (AR) [2], or AR-associated concepts, prior to receiving FDA marketing approval. The AR requires that products be tested in well-characterized animal models that are relevant to human disease and that the animal study endpoints be clearly related to the desired benefit in humans, generally the enhancement of survival or prevention of major morbidity. The cynomolgus monkey (*Macaca fascicularis*) NHP is the most widely used animal model for Ebola virus (EBOV) vaccine testing [3] and is well-characterized [4], although NHPs are currently in short supply [5]. Due to the high risk of aerosol transmission and life-threatening disease, filoviruses can only be handled safely using Biosafety Level 4 (BSL-4) practices. In order to use limited BSL-4 resources efficiently and to minimize NHP usage, a standard approach was adapted to efficiently screen candidate Ebola virus vaccines, formulations, and schedules.

Simon’s two-stage design was developed for “Phase II” cancer trials, which are one arm, uncontrolled trials (i.e., no statistical comparisons to controls) used to identify drugs with sufficient activity to warrant further development and for ethical reasons, typically are subject to early termination if there is low activity [6,7,8,9,10]. This approach has also been implemented in AIDs research [11] and gastroesophageal research [12]. A property of Simon’s two-stage design is that only a very small number of subjects may need to be tested to identify a poor candidate. For our screening program, this approach minimized the number of NHPs required to identify candidate vaccines that are most likely poorly protective (e.g., <50% effective), lowered the overall costs and permitted faster evaluation of the more promising candidate vaccines and vaccine regimens. Simon’s two-stage design was used to screen 27 EBOV vaccine candidates in 43 candidate regimens over a 5 year period under the National Institute of Allergy and Infectious Diseases (NIAID) Division of Microbiology and Infectious Diseases (DMID) Preclinical Services Vaccine Screening program. The EBOV vaccines approved by the EMA [13,14,15] and FDA [16] in 2019 were tested in this program. While there are now licensed vaccines for EBOV, there remains a need for safe and efficacious vaccines against Sudan virus (SUDV) and Marburg virus (MARV). The Simon’s two-stage screening strategy may prove useful for efficient screening of candidate vaccines for MARV and SUDV, as well as other MCM that are developed and tested under the AR.

## 2. Materials and Methods

### 2.1. Study Design

To assess potential designs with different Stage 1 and Stage 2 sample sizes and acceptance criteria for determining whether a candidate should be dropped or tested further, we computed the probabilities of accepting/rejecting candidates assuming the true effectiveness (probability of protection) of a vaccine is known and assuming the true outcome of the disease in the absence of the vaccine is death. That is, all animals in the challenge study will otherwise succumb to the EBOV challenge in a defined time frame following a defined disease course after exposure to a known dose (100 pfu) of the BSL4 site’s well characterized challenge material (FANG designated Ebola kikwit in all studies). The Type I (α) and Type II (β) error rates were used to compare various designs; however, we allowed mild deviations from the target values for some settings of protection to optimize a design that would work well over a broad range of protection.

We considered designs with high probability of correctly identifying adequately “effective” vaccines (with true protection rate of ≥80%) and high probability of correctly identifying “ineffective” vaccines (with true protection rate of ≤40%). Our goal was to identify those vaccines most likely to protect 80 percent or more of the NHP in our EBOV challenge model and to stop testing vaccines that do not meet this bar. We therefore focused designs towards finding the smallest Stage 1 sample size needed to identify ineffective vaccines with 80% or greater confidence. Table 1 provides probabilities of accepting effective or rejecting ineffective vaccine candidates for several designs that test up to ten NHPs in two stages, with up to four NHPs tested in Stage 1. Table 1 also shows the expected number of NHPs tested for a very ineffective vaccine (10% probability of protection); this number allows for the limited probability that a very ineffective candidate passes to Stage 2 testing. 

The design that minimizes NHPs used to eliminate ineffective vaccines and meets our goal of identifying effective vaccines and rejecting ineffective vaccines with at least 80 percent probability has a total of six NHPs, with two NHPs in Stage 1 and four in Stage 2. For a vaccine candidate that is minimally effective (80% efficacious), there is 88.5% probability it will be accepted based on the full testing in six NHPs. For a candidate vaccine at the top of the ineffective category (40% efficacious), there is 83% probability it will be rejected, often after testing the first two NHPs. For a vaccine that is very ineffective (10% efficacious), the expected number of NHPs tested is 2.76, which allows for the probability of advancing to Stage 2. This design was adopted as the optimal design.

**Figure 1:**
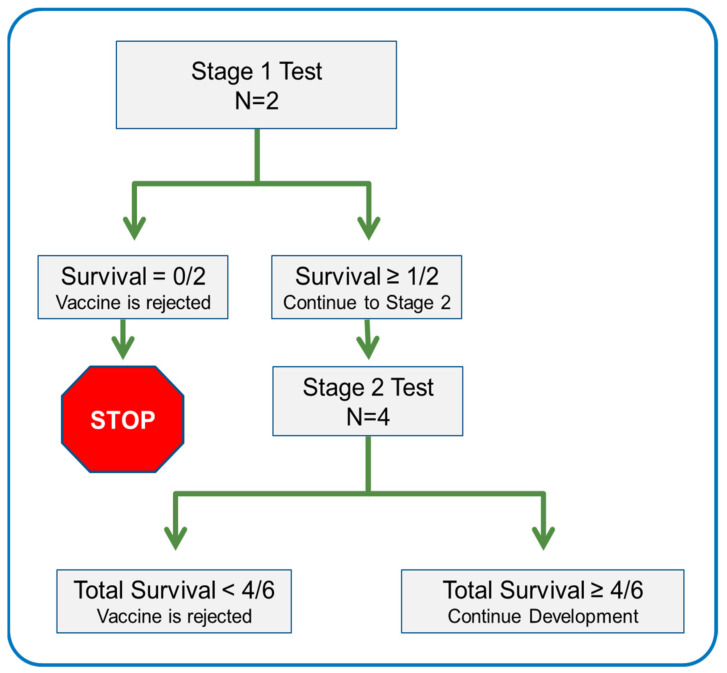
Decision tree for optimal Simon’s two-stage testing of vaccine candidates.

The optimal design was implemented as follows: Two NHPs were tested in Stage 1. If neither survived a normally lethal challenge, testing stopped for that candidate. If one or more NHPs survived, an additional four NHPs were tested in Stage 2. At most six NHPs were tested in two stages. A candidate vaccine could be rejected after either stage. A candidate vaccine was accepted for further investment/advancement if four or more NHPs were protected over both stages (Figure 1).

Alternate designs were used on a limited basis in the screening program. Eight tests were conducted using three or four NHPs in Stage 1 prior to adopting the optimal design. Later, five alternative regimens based on vaccine candidates that had been successfully tested in Stage 1 were tested using four NHPs in Stage 1. These candidate regimens were rejected if at most one of three NHPs survived or if at most two of four NHPs survived in Stage 1. As with the optimal design, at most six NHPs were tested, and the vaccine candidate was considered for advancement if four or more NHPs were protected over both stages. These designs also had greater than 80% probability that a minimally effective (80% efficacious) vaccine candidate would be accepted and greater than 80% probability that an ineffective vaccine candidate (40% efficacious) would be rejected, but the expected number of NHPs tested for ineffective vaccine candidates was greater than that for the optimal design (Table 1). Therefore, after implementing the optimal design with two NHPs in Stage 1, use of alternative designs was limited to modified regimens for vaccine candidates that had been successfully tested in Stage 1 using the optimal design.

### 2.2. Animal Model

Adult cynomolgus monkey (*Macaca fascicularis*) NHPs were used in the studies with ~50% males and females. Immunizations and blood draws were performed under ketamine anesthesia. Vaccinations were given at the indicated doses and vaccine composition in the quadriceps femoris, as a single injection with a volume of 0.5–1.0 mL. NHPs were transferred to the BSL-4 laboratory and acclimatized for approximately one week, then exposed to 100 pfu EBOV Kikwit as a single intramuscular injection in 0.5 mL volume. EBOV exposures were performed with Filovirus Animal Nonclinical Group (FANG) approved stocks originating from lethal human infections [17]. EBOV virus stocks were tested to be of identical sequence to wild-type viruses by deep sequencing and were shown to be endotoxin free. Animals were monitored at least twice daily after exposure and more frequently when clinical signs became apparent. Clinical observations varied between exposure facilities, but generally included responsiveness, severity and onset of rash, bleeding location and onset of bleeding, respiration, elevated body temperature, food consumption, signs of dehydration, stool, edema, and appearance of hair/coat. A clinical scoring system was used to monitor clinical signs of disease according to an Institutional Animal Care Use Committee approved scoring sheet for each institution. These clinical scores were used to assess whether the NHP met the criteria for humane termination [4,18].

## 3. Results

A total of 50 Stage 1 or Stage 2 tests of EBOV vaccine candidates were performed using Simon’s two-stage design with two, three, or four NHPs in Stage 1 and up to six total NHPs (Table 2), in nine studies conducted over a five-year period (2012–2017) under the NIAID/DMID Preclinical Services Vaccine Screening program. Each vaccine candidate was shown to be immunogenic in a rodent (mouse or guinea pig) model before initiating two-stage testing in NHPs [19,20,21,22,23,24,25]. Some were also shown to be efficacious in a rodent challenge model that used adapted virus [26]. None of the vaccine candidates were tested in NHP prior to entry into the screening program. In the screening program, the same vaccine candidate was sometimes tested in multiple candidate regimens with alternate formulations or schedules, which may have included with or without adjuvant, alternative adjuvants, monovalent or multivalent (made up of successful monovalent) formulations, single vaccination or homologous or heterologous prime boost schedules with varied timing of the boost(s). The 50 tests performed used 132 NHPs to evaluate 27 vaccine candidates in 43 candidate regimens that varied in dose, adjuvant, formulation, and/or schedule. The results are discussed for two groups of Stage 1 tests: (1) 30 candidate regimens tested using the optimal Simon’s two-stage design with two NHPs in Stage 1 and (2) thirteen candidate regimens tested using three or four NHPs in Stage 1. 

Thirty candidate regimens were screened in two NHPs in Stage 1 of Simon’s two-stage design. Applying the decision rule, 15 of these candidate regimens were eligible for Stage 2 testing in four additional NHPs and 15 were stopped based on zero of two survivors in Stage 1 (outcomes for treated NHP was the same as outcomes for untreated NHP). In practice, Stage 2 testing was not performed for every eligible vaccine candidate or regimen identified. For example, clinical signs were sometimes used in the survivors to distinguish among multiple formulations or different doses or schedules using the same vaccine candidate, so that only the optimal formulation or regimen was tested in Stage 2. Additionally, Stage 2 testing of a monovalent may not be completed when the corresponding multivalent was not efficacious in Stage 1. Nine of the 15 candidate regimens eligible for Stage 2 tests were withdrawn by the product sponsor without further testing. The remaining six candidate regimens were tested in Stage 2. All six candidate regimens qualified for advanced development. 

Of the 13 candidate regimens tested using three or four NHPs in Stage 1, eight were conducted prior to adoption of the optimal design and the remaining five tests utilized four NHPs based on a previous, successful Stage 1 test with two NHPs of the same candidate vaccine using a different regimen. These five tests were conducted concurrently with the Stage 2 test of the vaccine’s original regimen. Of the eight candidate regimens tested prior to adoption of the optimal design, candidate regimens were rejected in Stage 1 if one or none of three NHPs survived or if two or fewer of four NHPs survived. Otherwise, 2 or 3 additional NHPs could be tested with the candidate in Stage 2. As with the optimal design, at most six NHPs were tested, and the vaccine candidate was considered for advancement if four or more NHPs were protected over both stages. Four candidate regimens with two or three NHP survivors would have been eligible for testing in additional NHPs up to a total of six. One of these was tested in one additional NHP and passed into advanced development based on four survivors of four tested. One additional candidate regimen was advanced by the manufacturer without further testing in this program based on three survivors of three tested. The remaining two were withdrawn from testing.

Finally, five candidate regimens were initially tested in four NHPs based on a successful Stage 1 test of the candidate vaccine. These new candidate regimens varied in dose or schedule compared to the successful candidate regimen and were tested concurrently with the Stage 2 test of the successful candidate regimen. One candidate regimen qualified for advanced development with four of four survivors in Stage 1, one was eligible for testing in two additional NHPs but withdrawn from further testing, and three were rejected based one or two survivors in Stage 1.

## 4. Discussion

The BSL-4 laboratories available for this program had a maximum capacity of 16 to 24 NHPs per room. Two naïve NHPs were included in each screening study as process controls to verify that a lethal exposure was administered. Thus, seven to eleven Stage 1 candidates or three to five Stage 2 candidates could be tested in a single study within a single room. A total of 132 NHPs were used to test 27 vaccine candidates in 43 candidate regimens that varied in dose, adjuvant, formulation, or schedule. Fifteen of thirty candidate regimens tested in the optimal design stopped after Stage 1 due to low survival, and other factors were used to eliminate nine candidate regimens that were eligible for Stage 2 testing based on the decision rule. Therefore, a total of 24 candidate regimens were rejected or withdrawn based on testing in just two NHPs, thereby minimizing the number of NHPs tested for those regimens. Had Simon’s two-stage design not been implemented, these regimens would have been tested in at least 4 animals, which would have required 48 or more additional NHPs or, if additional animal or BSL4 facility resources were constrained or not available, would have reduced the number of candidate regimens that could be tested.

Eight candidate regimens were identified for advanced development in this program: one tested using four NHPs in two stages, six based on full Stage 1 and Stage 2 testing in six NHPs, and one tested in four NHPs in Stage 1 based on prior results for the vaccine candidate. In addition, one candidate regimen was advanced by the product developer based on testing in 3 NHPs. Multiple candidate regimens for USG-supported EBOV vaccines developed prior to and/or in response to the West African outbreak were tested and selected under this program for testing in humans, including the two-dose vaccine regimen, Zabdeno^®^ (Ad26.ZEBOV) and Mvabea^®^ (MVA-BN-Filo) from the Janssen Pharmaceutical Companies of Johnson & Johnson (EMA approved in 2020) [14,15] and the single dose vaccine regimen from Merck, Ervebo^®^ (FDA licensed in 2019 [16] and conditionally approved by the EMA in 2019, which switched to full approval in 2021 [13]). 

## 5. Conclusions

For many infectious diseases, the NHP serves as the model of choice for pre-clinical efficacy testing, especially when small animal models are either not available or only possible through the use of mouse or other species-adapted viruses. The selection of the most promising EBOV vaccine candidates to move forward in development was complicated by a large number of vaccine candidates developed to address the West African outbreak, the need to identify the optimal dose/schedule regimens for best efficacy, and the limited number of BSL4 facilities that can carry out the needed oversight of these challenge studies. As demonstrated in this case study for EBOV, the strategy to employ the Simon’s two-stage design for the initial screening of vaccine candidates significantly reduced the number of NHPs required for quickly identifying the most promising vaccine candidate(s) and regimens to move forward into the clinic. The success of Simon’s two-stage design for screening EBOV vaccine candidates suggests that this approach may prove useful for efficient screening of candidate vaccines for other filoviruses where the respective NHP challenge models are sufficiently characterized and virus challenge materials standardized to the extent possible across the BSL4 test sites [27]. 

## Figures and Tables

**Table 1:** Examples of two-stage screening designs that meet design objectives of greater than 80 percent probability of accepting an effective vaccine and greater than 80 percent probability of rejecting an ineffective vaccine.

Total NHPs (Stage 1 + Stage 2)	Total Survivors to Accept Vaccine (Stage 1 + Stage 2)	NHPs Tested Stage 1	Number of Survivors to Reject Vaccine after Stage 1	NHPs Tested Stage 2	Probability of Accepting 80% Effective Vaccine	Probability of Rejecting 40% Effective Vaccine	Expected Number of NHPs Tested for 10% Effective Vaccine
6	≥4	**2**	**0**	**4**	**0.885**	**0.830**	**2.76** ^1^
		3	≤1	3	0.852	0.848	3.08
		4	≤1	2	0.901	0.821	4.11
		4	≤2	2	0.803	0.876	4.01
7	≥5	2	0	5	0.839	0.907	2.95
		3	≤1	4	0.813	0.915	3.11
		4	≤1	3	0.852	0.904	4.16
		4	≤2	3	0.773	0.926	4.01
10	≥6	2	0	8	0.935	0.852	3.52
		3	≤1	7	0.881	0.879	3.20
		4	≤1	6	0.950	0.848	4.31
		4	≤2	6	0.812	0.910	4.02
10	≥7	2	0	8	0.859	0.948	3.52
		3	≤1	7	0.822	0.954	3.20
		4	≤1	6	0.872	0.947	4.31
		4	≤2	6	0.772	0.961	4.02

^1^ The optimal design (in bold) with six NHPs total and two NHPs in Stage 1 minimized the expected number of NHPs tested for an ineffective vaccine.

**Table 2:** Summary of EBOV vaccine candidate regimens tested under the NIAID/DMID preclinical services vaccine screening program between 2012 and 2017 using Simon’s two-stage design.

Design (Number Tested in Stage 1)	Testing Stage	Type of Vaccine	Adjuvant	Schedule	Number Tested ^1^	Total Survival ^1^	Next Step
Simon (4) ^2^	1	Monovalent	No	homologous prime boost	4	0	Stop
Simon (4) ^2^	1	Multivalent	No	homologous prime boost	4	1	Stop
Simon (4) ^2^	1	Multivalent	No	single dose	4	3	Eligible for Stage 2
Simon (3) ^2^	1	Monovalent	No	homologous prime boost	3	0	Stop
Simon (3) ^2^	1	Monovalent	No	homologous prime boost	3	2	Eligible for Stage 2
Simon (3) ^2^	1	Multivalent	Yes	homologous prime boost	3	0	Stop
Simon (3) ^2^	1	Monovalent	No	single dose	3	3	Eligible for Stage 2Manufacturer advanced
Simon (3) ^2^	1	Monovalent	No	single dose	3	3	Eligible for Stage 2
Simon (3) ^2^	2	Monovalent	No	single dose	3 + 1	3 + 1	Advance
Optimal Simon (2)	1	Monovalent	No	homologous prime boost	2	0	Stop
Optimal Simon (2)	1	Monovalent	Yes	homologous prime boost	2	1	Eligible for Stage 2
Optimal Simon (2)	1	Monovalent	Yes	homologous prime boost	2	0	Stop
Optimal Simon (2)	1	Monovalent	No	single dose	2	0	Stop
Optimal Simon (2)	1	Monovalent	Yes	single dose	2	0	Stop
Optimal Simon (2)	1	Monovalent	No	heterologous prime boost	2	2	Eligible for Stage 2
Optimal Simon (2)	1	Multivalent	No	heterologous prime boost	2	2	Eligible for Stage 2
Optimal Simon (2)	1	Multivalent	No	heterologous prime boost	2	2	Eligible for Stage 2
Optimal Simon (2)	1	Multivalent	No	heterologous prime boost	2	2	Eligible for Stage 2
Optimal Simon (2)	1	Multivalent	No	heterologous prime boost	2	2	Eligible for Stage 2
Optimal Simon (2)	1	Monovalent	Yes	homologous prime boost	2	1	Eligible for Stage 2
Optimal Simon (2)	1	Monovalent	Yes	homologous prime boost	2	0	Stop
Optimal Simon (2)	1	Monovalent	No	homologous prime boost	2	2	Eligible for Stage 2
Optimal Simon (2)	1	Monovalent	Yes	homologous prime boost	2	2	Eligible for Stage 2
Optimal Simon (2)	1	Monovalent	Yes	homologous prime boost	2	2	Eligible for Stage 2
Optimal Simon (2)	1	Monovalent	Yes	homologous prime boost	2	1	Eligible for Stage 2
Optimal Simon (2)	1	Monovalent	Yes	homologous prime boost	2	0	Stop
Optimal Simon (2)	1	Monovalent	Yes	homologous prime boost	2	0	Stop
Optimal Simon (2)	1	Multivalent	No	homologous prime boost	2	0	Stop
Optimal Simon (2)	1	Multivalent	No	heterologous prime boost	2	2	Eligible for Stage 2
Optimal Simon (2)	1	Monovalent	No	single dose	2	0	Stop
Optimal Simon (2)	1	Monovalent	Yes	homologous prime boost	2	0	Stop
Optimal Simon (2)	1	Monovalent	Yes	homologous prime boost	2	0	Stop
Optimal Simon (2)	1	Monovalent	Yes	homologous prime boost	2	2	Eligible for Stage 2
Optimal Simon (2)	1	Monovalent	Yes	homologous prime boost	2	2	Eligible for Stage 2
Optimal Simon (2)	1	Multivalent	Yes	homologous prime boost	2	1	Eligible for Stage 2
Optimal Simon (2)	1	Monovalent	Yes	homologous prime boost	2	0	Stop
Optimal Simon (2)	1	Monovalent	Yes	homologous prime boost	2	0	Stop
Optimal Simon (2)	1	Monovalent	Yes	homologous prime boost	2	0	Stop
Optimal Simon (2)	1	Monovalent	Yes	homologous prime boost	2	0	Stop
Optimal Simon (2)	2	Multivalent	No	heterologous prime boost	2 + 4	2 + 3	Advance
Optimal Simon (2)	2	Multivalent	No	heterologous prime boost	2 + 4	2 + 3	Advance
Optimal Simon (2)	2	Multivalent	No	heterologous prime boost	2 + 4	2 + 4	Advance
Optimal Simon (2)	2	Multivalent	No	heterologous prime boost	2 + 4	2 + 2	Advance
Optimal Simon (2)	2	Monovalent	Yes	homologous prime boost	2 + 4	1 + 3	Advance
Optimal Simon (2)	2	Monovalent	Yes	homologous prime boost	2 + 4	2 + 3	Advance
Simon (4) ^3^	1	Monovalent	Yes	homologous prime boost	4	2	Stop
Simon (4) ^3^	1	Multivalent	No	heterologous prime boost	4	4	Advance
Simon (4) ^3^	1	Multivalent	No	heterologous prime boost	4	2	Stop
Simon (4) ^3^	1	Multivalent	No	heterologous prime boost	4	3	Eligible for Stage 2
Simon (4) ^3^	1	Multivalent	No	heterologous prime boost	4	1	Stop

^1^ For or Stage 1 test, the number in Stage 1. For Stage 2 test, the numbers in Stage 1 + Stage 2. ^2^ Stage 1 test conducted with three or four NHPs prior to adopting the optimal design. ^3^ Stage 1 test of an alternate regimen of a vaccine candidate that had passed Stage 1 test using the optimal design, tested concurrently with the Stage 2 test of the original regimen.

## Data Availability

All relevant data provided in Table 2.
